# Efficacy and safety of misoprostol compared with dinoprostone for labor induction at term: an updated systematic review and meta-analysis of randomized controlled trials

**DOI:** 10.3389/fmed.2024.1459793

**Published:** 2024-12-09

**Authors:** Nusrat Lakho, Mahrukh Hyder, Taimoor Ashraf, Sajida Khan, Ajay Kumar, Maheen Jabbar, Madhurta Kumari, Asfia Qammar, Sateesh Kumar, Muskan Kumari, Fnu Deepak, Kapil Raj, Azzam Ali

**Affiliations:** ^1^Isra University Karachi-Campus, Karachi, Pakistan; ^2^Jinnah Post Graduate Medical Centre, Karachi, Pakistan; ^3^Nishtar Medical College, Multan, Pakistan; ^4^Sri Aurobindo University, Indore, India; ^5^Shaheed Mohtarma Benazir Bhutto Medical University, Larkana, Pakistan; ^6^Bahria University Medical and Dental College, Karachi, Pakistan; ^7^Dow University of Health Sciences, Karachi, Pakistan

**Keywords:** misoprostol, dinoprostone, intravaginally, labor induction, term

## Abstract

**Background:**

Labor induction is a common obstetric intervention, increasingly performed worldwide, often using prostaglandins like misoprostol and dinoprostone.

**Objective:**

This study aims to compare the effectiveness and safety of intravaginal misoprostol versus dinoprostone for inducing labor, examining their impact on various maternal and neonatal outcomes.

**Methods:**

A systematic review and meta-analysis were conducted using four databases—PubMed, Google Scholar, EBSCO, and the Cochrane Library—from January 2000 to April 2023. We included randomized controlled trials (RCTs) involving singleton pregnancies at term (37–42 weeks) with unfavorable cervices, where intravaginal misoprostol was compared to dinoprostone. Key outcomes evaluated for effectiveness included vaginal delivery within 24 h, overall vaginal delivery rate, and need for oxytocin augmentation. Safety outcomes assessed were tachysystole, uterine hyperstimulation, abnormal cardiotocography, NICU admissions, cesarean delivery, and APGAR scores. Risk ratios (RRs) and 95% confidence intervals (CIs) were calculated using a random-effects model in Review Manager (RevMan) version 5.4.1.

**Results:**

Eight RCTs with a total of 1,801 participants (937 in the misoprostol group and 864 in the dinoprostone group) met the inclusion criteria. Misoprostol required a significantly less oxytocin augmentation than dinoprostone [RR = 0.83; 95% CI (0.71, 0.97), *p* = 0.02]. Other outcomes, including rates of cesarean delivery, uterine tachysystole, hyperstimulation, and NICU admissions, showed no significant differences between the two groups, indicating comparable safety and efficacy profiles.

**Conclusion:**

This meta-analysis demonstrates that intravaginal misoprostol is an effective and safe alternative to dinoprostone for labor induction at term. Misoprostol achieved comparable efficacy and safety outcomes while requiring less oxytocin augmentation, supporting its potential as a practical induction agent in clinical settings.

## Introduction

The delivery of a fetus can be induced by initiating intrauterine contractions using pharmacological or mechanical methods (1). Approximately 20% of all births are now intentionally induced through induction of labor (IOL), an increasingly common obstetric practice in modern obstetrics (2, 3), aimed at enhancing maternal and neonatal outcomes, especially when spontaneous labor may present risks. Common indications for labor induction include prolonged pregnancy (post-term), maternal conditions (e.g., hypertension, diabetes), and concerns about fetal well-being (e.g., intrauterine growth restriction) (4). Risks of stillbirth or neonatal death increase as gestation continues beyond term (around 40 weeks’ gestation), making timely induction a preventive measure (5). Evidence suggests that elective induction at 41 weeks—or potentially earlier under specific conditions—may lower the risks associated with cesarean delivery and complications like meconium-stained amniotic fluid (6). Labor induction success is often defined as achieving vaginal delivery within 24–48 h (7).

In recent years, the use of labor induction (IOL) has significantly increased, growing from 9.0% of all births in 1989 to 23% in 2012 (8). A 2012 study analyzing data from numerous hospitals across the United States discovered that over two-fifths (42.9%) of nulliparous women and slightly more than a third (31.8%) of multiparous women underwent labor induction (9). Pharmacological therapies, such as oxytocin and prostaglandins, are administered orally, vaginally, or intravenously to mature the cervix for labor induction. While oxytocin is effective for labor augmentation in women with favorable cervices, a ripening agent may be used when induction of labor is performed on women with unfavorable cervices (10–12). Other treatments designed to aid the induction process in cases of unfavorable cervix, such as membrane rupture, have been associated with reduced efficiency and higher failure rates (13).

Dinoprostone, a prostaglandin E2 analog, has traditionally been used to induce labor using either an intracervical gel or a vaginal insert (14). However, its use in resource-constrained settings is hindered by challenges such as cost and the requirement for cold storage (15). Misoprostol, a prostaglandin E1 analog originally used in the 1980s to manage and cure peptic ulcer disease (16), has been extensively studied in randomized clinical trials for its efficacy in gynecologic and obstetric procedures. It is utilized for inducing uterine contractions and cervical ripening to facilitate labor induction (17–19). Unlike dinoprostone, misoprostol is significantly more affordable, easier to administer, does not require cold storage, and is readily available even in resource-constrained countries, giving it a distinct advantage over dinoprostone (20).

Many clinical trials have investigated the effectiveness and safety of intravaginal misoprostol versus dinoprostone (17–19, 21–25), finding that misoprostol is more effective in minimizing the requirement for oxytocin augmentation in labor induction (26, 27). The meta-analysis conducted by Wang et al. (19) found comparable outcomes between the misoprostol and dinoprostone groups, showing no significant differences. However, it should be noted that their study included Saxena et al. (28) and Chitrakar et al. (29), who administered dinoprostone intracervically instead of vaginally, which goes against the specified inclusion criteria.

Considering these concerns, we conducted an updated meta-analysis to explore whether there are significant differences in various outcomes between the misoprostol and dinoprostone groups, contrasting with the nonsignificant findings reported by Wang et al. (19).

## Methods

This meta-analysis was conducted in accordance with the Preferred Reporting Items for Systematic Review and Meta-Analysis (PRISMA) guidelines (30).

### Literature search

A thorough search of PUBMED, Google Scholar, Ebsco, Cochrane Library, and CNKI was conducted from January 2000 to April 2023. The following combination of Medical Subject Heading (MeSH) terms and keywords were used in the database searches: “Misoprostol,” “Dinoprostone,” “Labor Induction,” “Intravaginally,” and “Term.” A detailed search strategy is presented in the Supplementary Table S1. Two independent reviewers thoroughly reviewed the titles, abstracts, full texts, and bibliographies of all identified studies separately to identify potentially relevant research. The assessment included a detailed examination of references in the relevant literature to identify appropriate studies, with no restrictions based on geographical location, ethnicity, or publication language. In cases of discrepancy, a third author was consulted to reach a consensus. Additionally, gray literature sources were searched to identify potential publications relevant to this study. A detailed search strategy is presented in the Supplementary Table S1.

### Data extraction

Initially, two reviewers independently examined the titles and abstracts of publications that met the inclusion criteria, followed by a comprehensive review of the full texts. Subsequently, they extracted data from the eligible studies and documented it in an information extraction table. Two researchers independently collected the following information from each study included in the analysis: (a) the name and year of the study, (b) study design, (c) study location, (d) the number of patients in each group (misoprostol vs. dinoprostone), (e) general characteristics of the patients (age, gestational weeks, dosage, and mean birth weight), and (f) all outcomes of interest. Any discrepancies in data extraction were resolved through discussion or by consulting a third reviewer.

### Inclusion and exclusion criteria

This study included only RCTs and adhered to strict eligibility criteria for research inclusion, with no restrictions on intervention dosage. The specific parameters are detailed below:

#### PICO

##### P: Population

Singleton pregnant women with live intrauterine gestations, unfavorable cervices, and a gestational period of 37 to 42 weeks.

##### I: Intervention

Intravaginal misoprostol.

##### C: Comparison

Intravaginal dinoprostone.

##### O: Outcome

Cesarean section rate, vaginal delivery rate, vaginal delivery within 24 h, incidences of uterine tachysystole (defined as at least six contractions in a 10-min period sustained over two consecutive 10-min intervals), hyperstimulation (defined as fetal heart rate abnormality associated with tachysystole), necessity for oxytocin augmentation, NICU admissions, abnormal cardiotocography readings, and APGAR scores below 7 at 5 min.

Studies were excluded for various reasons, including unsuitable design (such as non-randomization), lack of relevant data, involvement of animal models, or if they were case reports, editorials, reviews, conference abstracts, or duplicate publications.

### Statistical analysis

Statistical analysis was conducted using Review Manager (RevMan) version 5.4.1, following The Cochrane Collaboration’s (2020) guidelines. For pooling categorical outcomes, risk ratios (RRs) and their corresponding 95% confidence intervals (CIs) were calculated using a random-effects meta-analysis approach. A random-effects meta-analysis was also performed for continuous outcomes to determine mean differences (MDs) and their 95% confidence intervals (CIs). Sensitivity analysis was conducted to address outcomes with severe heterogeneity. Funnel plots were not generated due to the presence of fewer than 10 studies. Higgins’ I^2^ statistics were used to quantify heterogeneity: I^2^ values of 25–50% indicated mild heterogeneity, 50–75% indicated moderate heterogeneity, and values greater than 75% indicated severe heterogeneity (31). To identify and address sources of heterogeneity, sensitivity analyses were planned to use the leave-one-out method. A *p*-value of 0.05 or less was considered statistically significant.

### Risk of bias assessment

The risk of bias within individual studies was evaluated using the Cochrane Risk of Bias Tool, which examines potential sources of bias across multiple domains, including random sequence generation, allocation concealment, blinding of participants and personnel, blinding of outcome assessment, incomplete outcome data, selective reporting, and other potential sources of bias. The risk of bias in each category was systematically classified as low, high, or unclear (32).

## Results

### Study selection, baseline, and characteristics overview

**Figure 1:**
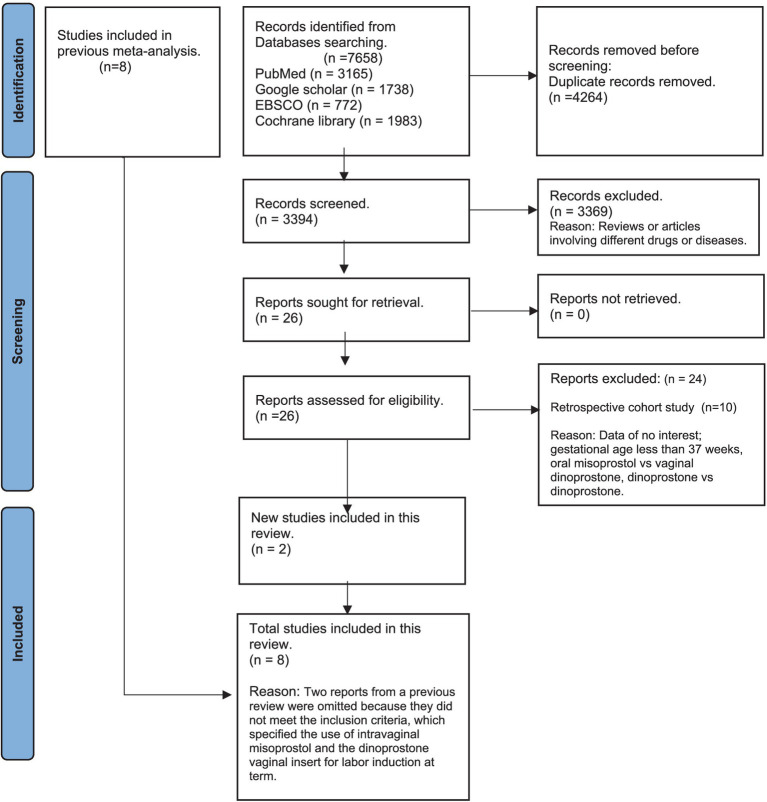
PRISMA flow chart.

From an initial 7,658 search results across PubMed, Google Scholar, Cochrane Library, and EBSCO, we removed 4,264 duplicates, leaving 3,394 studies for screening. After excluding irrelevant studies, 26 full-text articles were reviewed. A full-text review of 26 studies followed, leading to the exclusion of 10 cohort studies (25, 33–41) and 14 studies due to non-relevant data, gestational age under 37 weeks, or inappropriate comparisons (21, 22, 42–53). Ultimately, eight RCTs were included in the analysis—six from Wang et al.’s meta-analysis (19) and two newly identified RCTs meeting our criteria (Figure 1).

Table 1 details the baseline and study characteristics of the included trials. This analysis encompasses eight RCTs with a total of 1,801 participants. Of these, 937 received misoprostol, while 864 were in the dinoprostone group. The dosage and administration regimens for both drugs varied among the trials. In three studies (17, 54, 55), the misoprostol group received 25 micrograms (μg) every 4 h for a total of six doses. One trial administered up to five doses of 50 μg every 4 h (56), two trials gave up to two doses of 25 μg every 6 h (18, 57), two trials administered up to three doses of 50 μg every 6 h (58, 59), and another trial administered up to two doses of 50 μg every 6 h (57).

**Table 1:** General and baseline characteristics of the studies.

Study	Patient population	Country	Year	Misoprostol	Dinoprostone	Mean age years (*m* ± SD)		Dosage		Mean period of gestational weeks (m ± SD)		Mean Bishop score at induction (*m* ± SD)		Mean birth weight (grams ± SD)	
						Misoprostol	Dinoprostone	Misoprostol	Dinoprostone	Misoprotol	Dinoprostone	Misoprostol	Dinoprostone	Misoprostol	Dinoprostone
Young et al. (18)	344	Canada	2020	172	172	28.8 (± 5.6)	29.1 (± 5.70)	25 μg every 6 h	1-2 mg every 6 h	39.4 (±1.4)	40.0(±1.5)	4.1 (±1.9)	4.2 (±2.1)	3,621 (±557)	3,598 (±530)
De Bonrostro Torralba et al. (17)	198	Spain	2019	99	99	33.52 (± 5.04)	33.49 (± 4.9)	25 μg every 4 h	10 mg	292 (291–292)	292 (292–292)	3 (2–3)	3 (2–4)	3482.92 (± 366.8)	3475.31 (± 359.0)
Gregson et al. (54)	268	United Kingdom	2005	139	129	28.73 (± 5.34)	29.57 (± 5.19)	25 μg every 4 h	1–2 mg every 6 h	289.02 (24.91)	290.31 (9.52)	NA	NA	3,720 (445)	3,819 (472)
Ozkan et al. (56)	112	Turkey	2009	56	56	NA	NA	50 μg in the posterior fornix every 4 h	10 mg vaginal insert for a maximum of 12 h	NA	NA	NA	NA	3,250 (± 519)	3,119 (± 622)
Prager et al. (55)	390	Sweden	2008	199	191	32.2	33.3	25 μg every 4 h	2 mg 6–8 h	40.3	40.2	NA	NA	3,702	3,693
Ayaz et al. (58)	120	Pakistan	2010	60	60	23	25	50 μg every 6 h	3 mg every 6 h	NA	NA	NA	NA	3,165 (± 430)	3,273 (± 390)
Tan et al. (57)	169	Singapore	2010	54	57	31.27 (± 5.38)	31.42 (± 5.19)	25 μg every 6 h	3 mg every 6 h	39.66 (±1.20)	39.38 (±1.35)	2.84 (±1.02)	2.70 (±0.96)	NA	NA
				58	29.95 (± 4.43)	50 μg every 6 h	39.65 (±1.26)	2.57 (±1.06)	NA	NA
Saeed et al. (59)	200	Pakistan	2009	100	100	26.22 (± 3.40)	26.22 (± 3.40)	50 μg every 6 h	3 mg every 6 h	40.11 (± 1.37)	40.11 (± 1.37)	3.12 (± 1.28)	NA	NA	NA

(m ± SD): mean and standard deviation; μg: microgram; mg: milligram.Note: mentions of Torralba et al. in any figure in the article refer to De Bonrostro Torralba et al. (17).

For the dinoprostone groups, two trials administered 1–2 milligrams (mg) every 6 h for 24 h (18, 54), one trial administered 2 mg for a maximum of four doses every 6 h (55), two trials administered a 10 mg vaginal insert for up to 12 h (17, 56), one trial administered 3 mg into the posterior vaginal fornix for up to two doses every 6 h (57), and two trials administered 3 mg into the posterior vaginal fornix for up to three doses every 6 h (58, 59).

### Quality assessment

**Figure 2:**
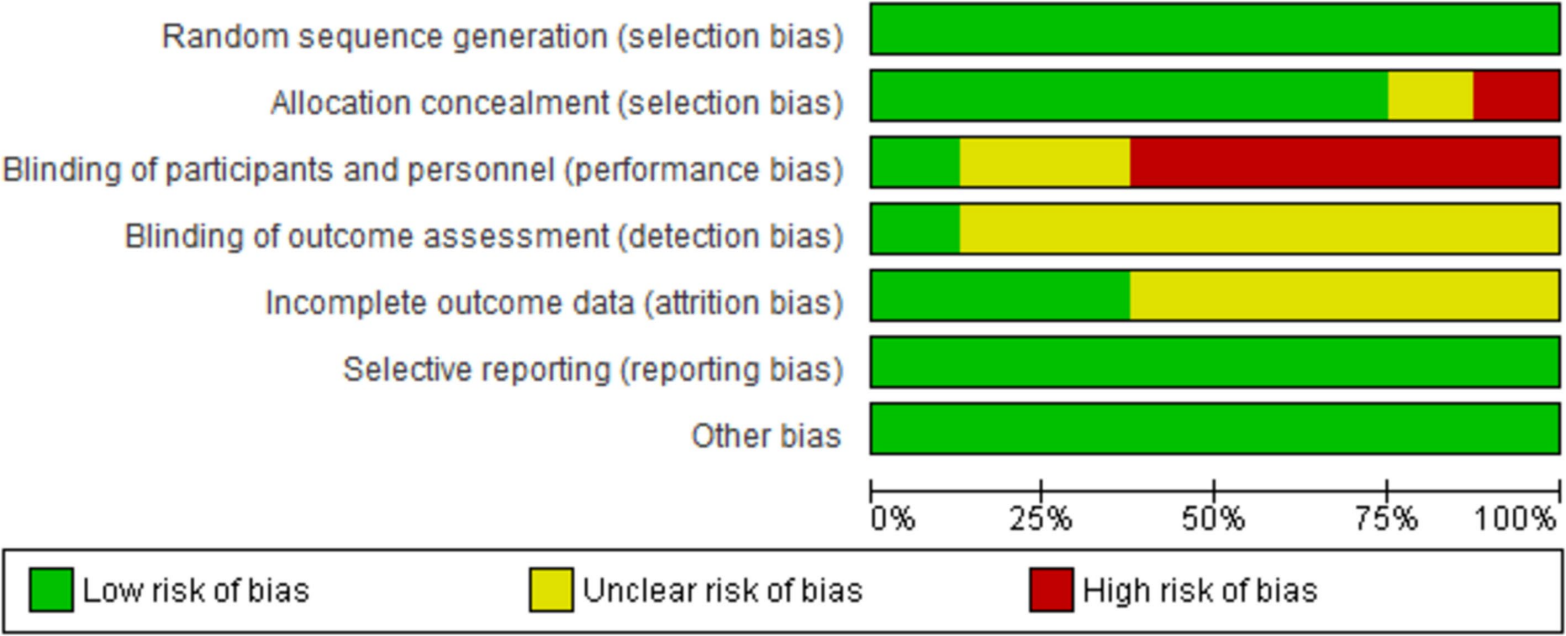
Risk of bias graph.

**Figure 3:**
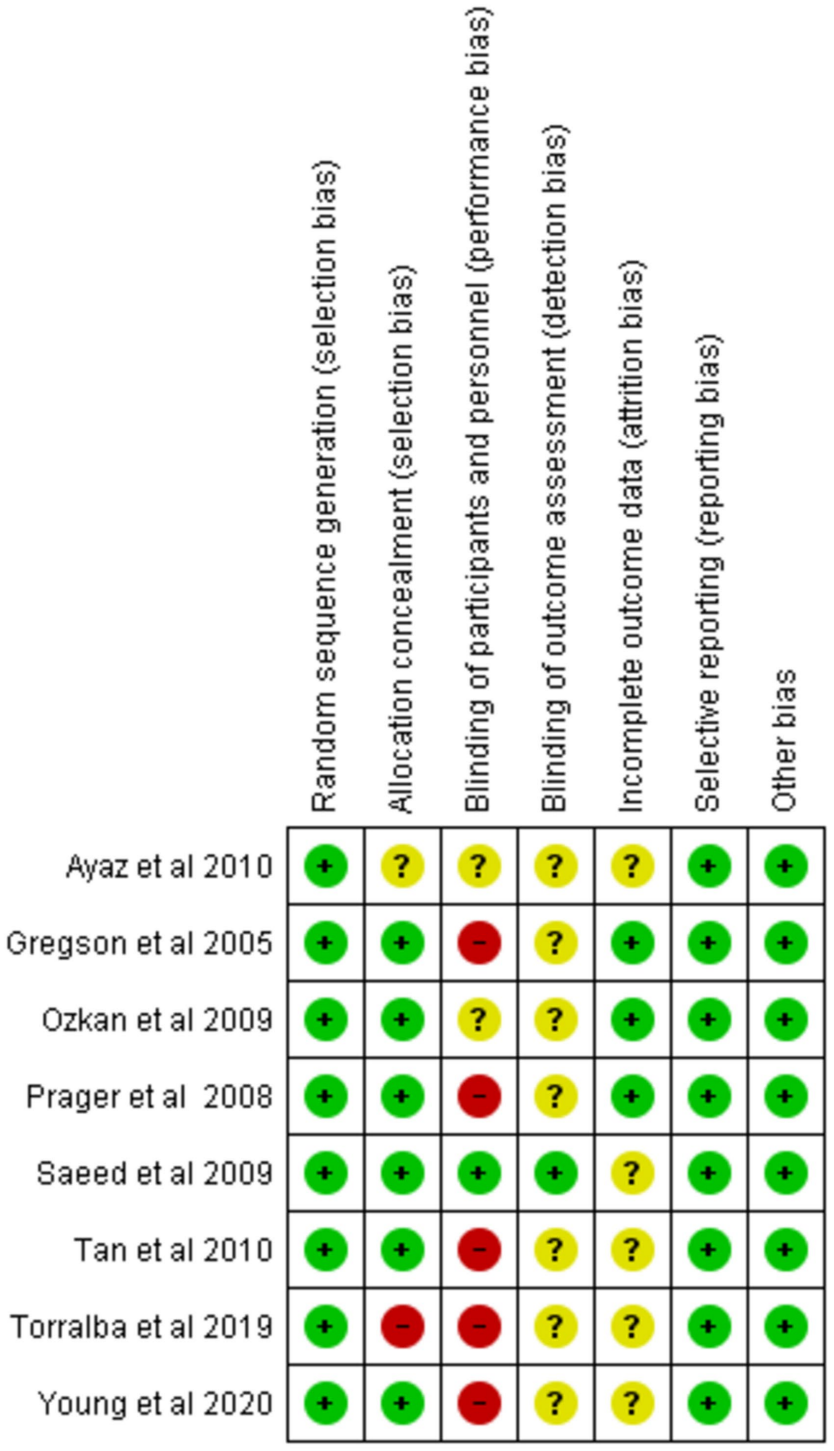
Risk of bias summary.

We evaluated the validity of the eight RCTs using the Cochrane Risk of Bias Tool. Overall, these studies were determined to be of excellent quality and exhibited a low risk of bias across all seven assessment categories, thereby enhancing the credibility of our findings. A comprehensive assessment is illustrated in Figures 2, 3.

### Maternal outcomes

#### Vaginal delivery within 24 h

**Figure 4:**
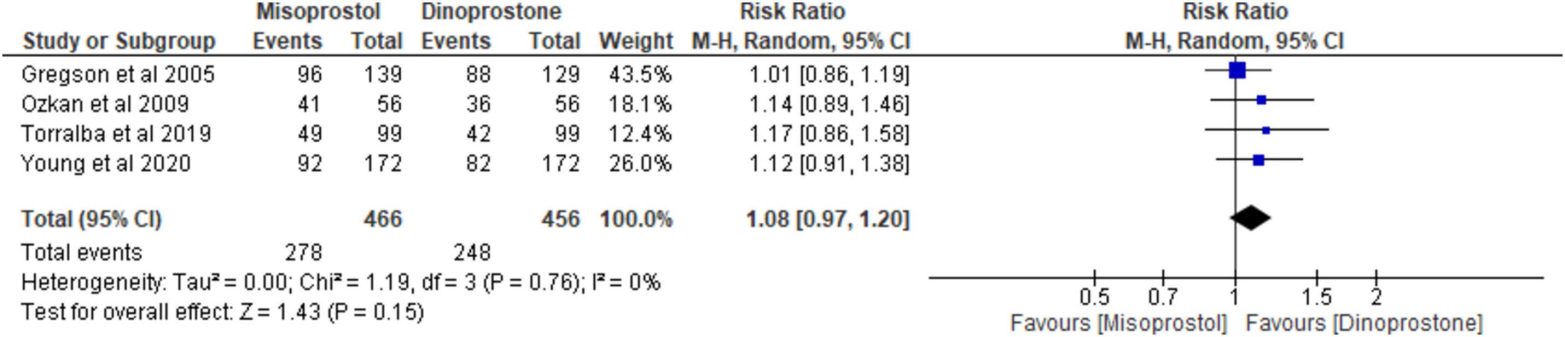
Forest plot of vaginal delivery at less than 24 h.

Four studies (17, 18, 54, 56) involving 922 patients reported on vaginal delivery within 24 h. Using a random-effects model to pool the results, no significant difference was found between misoprostol and dinoprostone in achieving vaginal delivery within 24 h [RR = 1.08; 95% CI (0.97, 1.20) *p* = 0.15]. Additionally, no statistically significant heterogeneity was observed among the studies (*p* = 0.76, *I*^2^ = 0%; Figure 4).

#### Cesarean delivery

**Figure 5:**
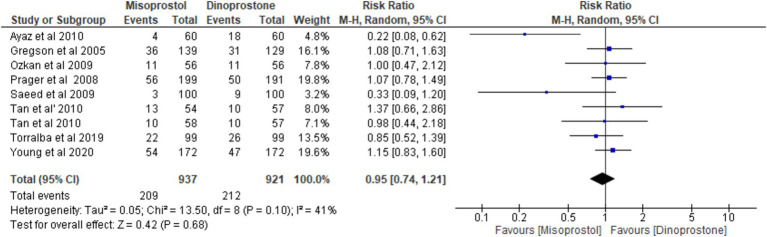
Forest plot of cesarean delivery.

Eight studies (17, 18, 54–59), encompassing 1,858 patients, reported on cesarean delivery. Using a random-effects model to pool the combined effects, the results indicated no significant difference between the misoprostol and dinoprostone groups [RR = 0.95; 95% CI (0.74, 1.21) *p* = 0.68]. Additionally, there was no significant heterogeneity among the studies (*p* = 0.10, *I*^2^ = 41%; Figure 5).

#### Oxytocin augmentation

**Figure 6:**
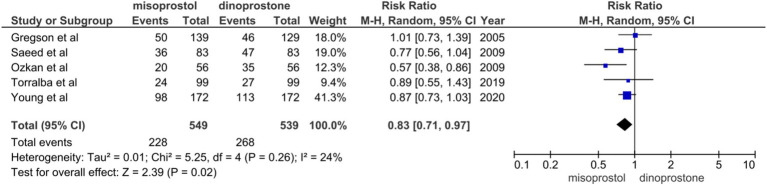
Forest plot of oxytocin augmentation.

Five studies (17, 18, 54, 56, 59) involving 1,088 patients reported on oxytocin augmentation. Using a random-effects model to pool the combined effect, the results showed that the misoprostol group required significantly less oxytocin compared to the dinoprostone group [RR = 0.83; 95% CI (0.71, 0.97) *p* = 0.02]. Additionally, there was no significant heterogeneity observed among the studies (*p* = 0.26, *I*^2^ = 24%; Figure 6).

#### Uterine tachysystole

**Figure 7:**
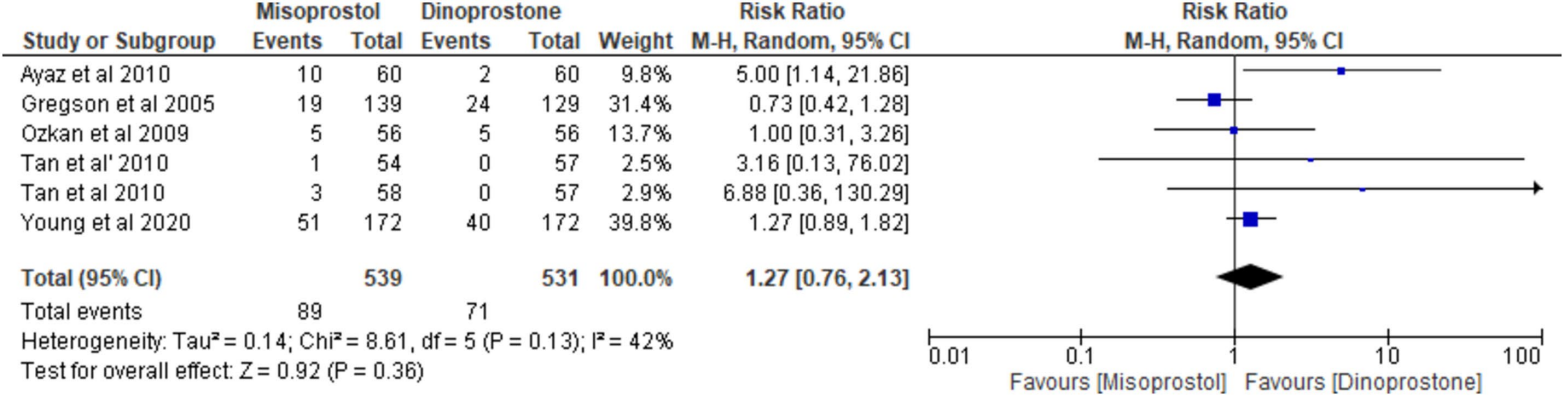
Forest plot of tachysystole.

Five studies (18, 54, 56–58) involving 1,070 patients reported on the incidence of tachysystole. Using a random-effects model to pool the combined effect, the results showed that misoprostol was not significantly associated with a higher incidence of tachysystole compared to dinoprostone [RR = 1.27; 95% CI (0.76, 2.13) *p* = 0.36]. Additionally, no significant heterogeneity was observed among the studies (*p* = 0.13, *I*^2^ = 42%; Figure 7).

#### Vaginal delivery

**Figure 8:**
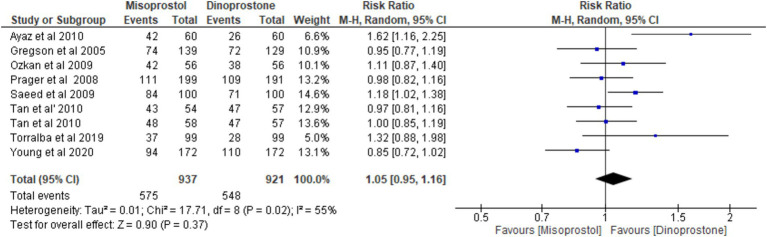
Forest plot of vaginal delivery.

Eight studies (17, 18, 54–59) involving 1,858 patients reported on vaginal delivery outcomes. Using a random-effects model to pool the combined effect, the results indicated no significant difference in vaginal delivery rates between misoprostol and dinoprostone [RR = 1.05; 95% CI (0.95, 1.16) *p* = 0.37]. Moderate heterogeneity was observed among the studies (*p* = 0.02, *I*^2^ = 55%; Figure 8).

#### Instrumental delivery

**Figure 9:**
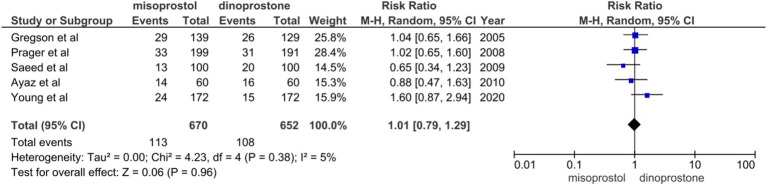
Forest plot of instrumental delivery.

Five studies (14, 48, 49, 52, 53) involving 1,322 patients reported on instrumental delivery. A random-effects model was used to pool the combined effect, revealing no significant difference between the misoprostol and dinoprostone groups [RR = 1.01; 95% CI (0.79–1.29) *p* = 0.96]. Furthermore, no notable heterogeneity was detected among these studies (*I*^2^ = 5%; Figure 9).

### Obstetrics outcomes

#### NICU admission

**Figure 10:**
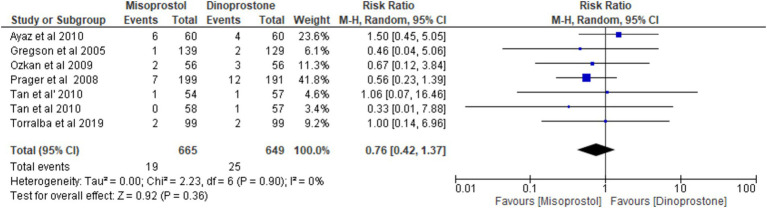
Forest plot of APGAR score < 8 at 5 min.

**Figure 11:**
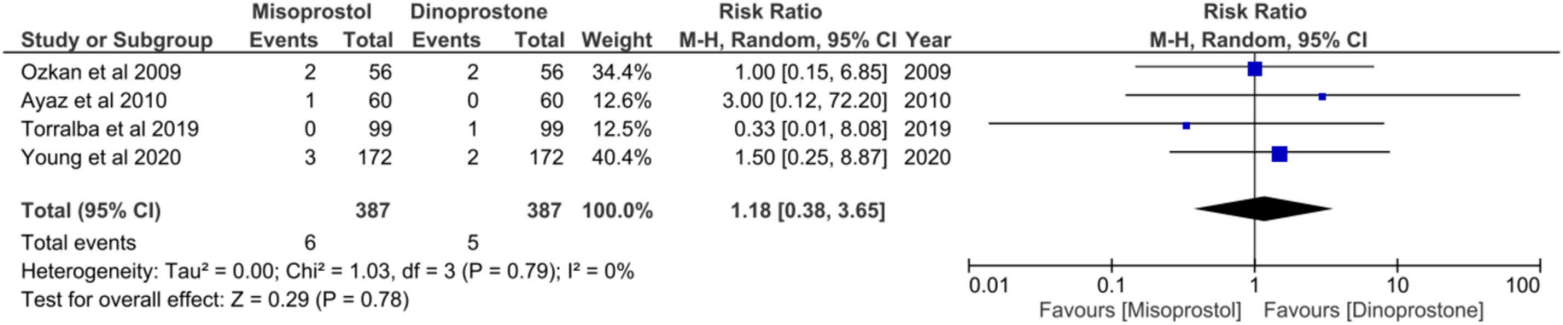
Forest plot of NICU admission.

Six studies (17, 54–58), encompassing a total of 1,314 patients, reported on the incidence of NICU admissions. Employing a random-effects model to synthesize the data, the pooled results indicated no statistically significant difference between the two cohorts [RR = 0.76; 95% CI (0.42, 1.37) *p* = 0.36] (Figure 10). Furthermore, no notable heterogeneity was detected among these studies (*p* = 0.90, *I*^2^ = 0%; Figure 11).

#### APGAR score < 8 at 5 min

Four studies (17, 18, 54, 58), comprising a total of 774 patients, reported on APGAR scores below 8 in 5 min. No statistically significant heterogeneity was observed among these studies (*p* = 0.79, *I*^2^ = 0%). A random-effects model was employed to aggregate the combined effect, demonstrating no significant difference in the incidence of APGAR scores below 8 at 5 min between the two groups [RR = 1.18; 95% CI (0.38, 3.65) *p* = 0.78] (Figure 10).

#### Abnormal cardiotocograph

**Figure 12:**
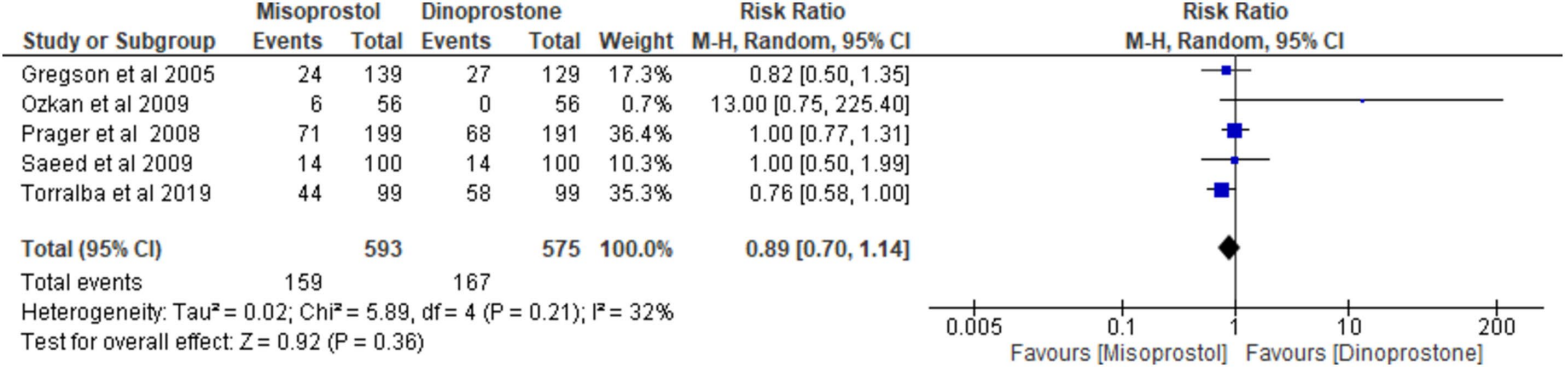
Forest plot of abnormal cardiotocograph.

Five studies (17, 54–56, 59), encompassing 1,168 patients, reported on the incidence of abnormal cardiotocograph results. Using a random-effects model to pool the combined results, the analysis revealed no significant difference between misoprostol and dinoprostone [RR = 0.89; 95% CI (0.70), 1.14; *p* = 0.36]. No statistically significant heterogeneity was observed among the studies (*p* = 0.21, *I*^2^ = 32%; Figure 12).

#### Hyperstimulation

Eight studies (17, 18, 54–59), involving 1858 patients, reported the incidence of uterine hyperstimulation. A random-effects model was used to pool the combined effect, which indicated no significant difference between misoprostol and dinoprostone [RR = 1.14; 95% CI (0.73, 1.79) *p* = 0.56]. No significant heterogeneity was observed among the studies (*p* = 0.37, *I*^2^ = 8%).

#### Leave-one-out analysis

No outcomes exhibited significant heterogeneity except for vaginal delivery. The leave-one-out sensitivity analysis revealed that the rate of vaginal delivery was influenced by a single study, namely Ayaz et al. (58). Excluding this study led to a notable reduction in *I*^2^ values (*p* = 0.16; *I*^2^ = 34%) and altered the overall effect [RR = 1.01, 95% CI (0.93, 1.10), *p* = 0.74] (Supplementary Figure S1).

## Discussion

**Figure 13:**
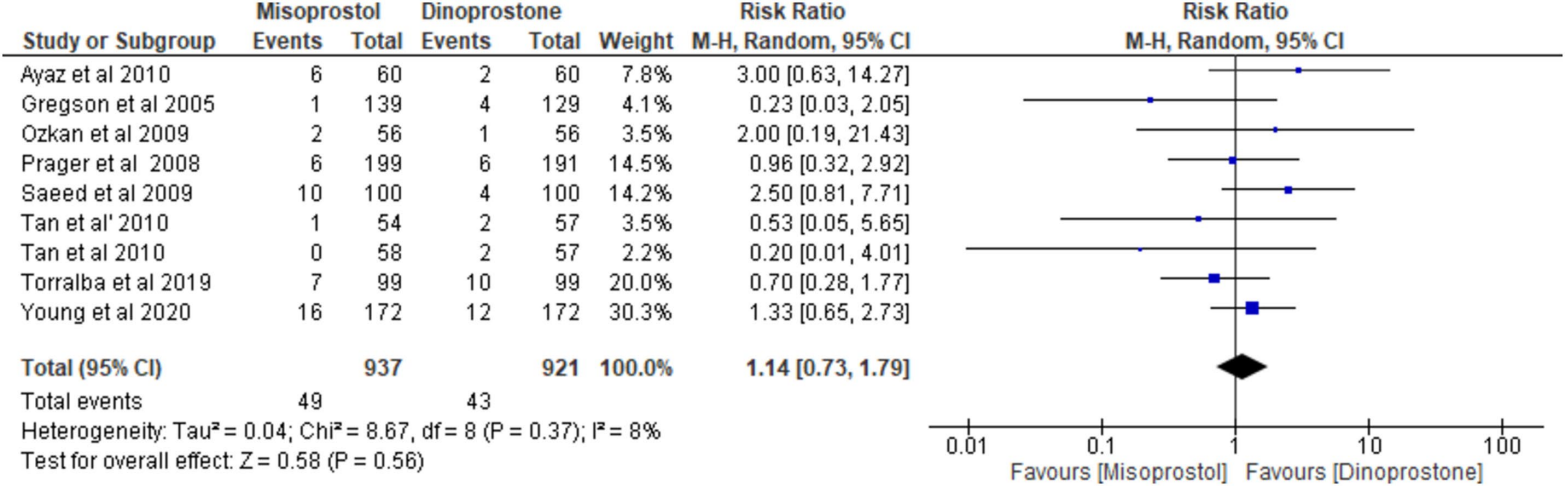
Forest plot of hyperstimulation.

This meta-analysis of eight RCTs comparing intravaginal misoprostol and dinoprostone for labor induction in women with unfavorable cervices at term found no significant differences between the two groups in key maternal and neonatal outcomes, such as vaginal delivery within 24 h, cesarean delivery, and overall vaginal delivery rates. While oxytocin augmentation was needed less frequently in the misoprostol group, other outcomes—including the incidence of uterine tachysystole, hyperstimulation, NICU admissions, low APGAR scores, and abnormal cardiotocograph readings—showed no notable differences between groups (Figure 13).

Wang et al.’s (19) study showed non- significant result in terms of oxytocin augmentation for dinoprostone group with a p value of (*p* = 0.11), while our meta-analysis clearly showed the significance need of oxytocin augmentation in dinoprostone group with a p value of (0.02). This could be attributed to the inclusion of two studies in the meta-analysis by Wang et al. (19) not aligning with inclusion criteria. This finding is supported by a study conducted by Meyer et al. (27) which states that misoprostol decreased the dose of oxytocin. Another meta-analysis by Liu et al. (60) comparing intravaginal misoprostol to intracervical dinoprostone concluded that the misoprostol group required less oxytocin augmentation than the dinoprostone group.

According to our findings, there were no appreciable changes in the two groups’ rate of Cesarean sections, which is consistent with meta-analysis by Wang et al. (19) and study by Wing et al. (61) that have reported inconsistent results regarding the impact of misoprostol on Cesarean section rates. Similarly, a study by Moodley et al. (62) also supports our findings by suggesting that neither intervention affects the rate of C-sections. Regarding vaginal delivery within 24 h, our results showed no significant difference, which is consistent with an observational study conducted by Moodley et al. (62). This lack of difference may be attributed to both interventions being equally efficient in promoting vaginal delivery. In addition to this, our study found out that there was no significant difference in instrumental delivery between the two groups. A recent comparative study by Sire et al. (63) aligns with the results of our analysis. However, a study conducted by Akhtar et al. (64) at a hospital in Pakistan shows that there is difference between two groups and use of dinoprostone shows greater incidence of instrumental delivery which could possibly be due to small sample size of the study.

Furthermore, this meta-analysis did not find a significant difference in hyperstimulation between the misoprostol and dinoprostone groups. This aligns with a randomized controlled trial conducted by Madaan et al. (65) which also found no significant difference between the two groups. In terms of neonatal outcomes, our study did not find any significant differences in NICU admissions, abnormal cardiotocographs, or APGAR scores below 7. These findings align with a previous meta-analysis conducted by Wang et al. (19). Another randomized controlled trial by Wing et al. (53) comparing dinoprostone vaginal insert with vaginal misoprostol insert also found no association between neonatal outcomes in treatment groups.

When compared to women who received dinoprostone treatment, women treated with misoprostol had a significantly lower rate of oxytocin augmentation (17, 18, 41, 54–59). This shows that misoprostol might be more efficient at accelerating the course of labor, hence minimizing the requirement for additional interventions. On the other hand, although not statistically significant, the occurrence of tachysystole was higher in women administered misoprostol (18, 54, 56–58). This may suggest that misoprostol may raise the incidence of tachysystole and could perhaps suggest that lower doses must be administered which calls for additional research. Furthermore, a review by Boulvain et al. (66), comparing misoprostol to other controls, also supports this association, suggesting that misoprostol is linked to uterine tachysystole. Additionally, Farah et al. (67) found that a higher dose of 50 μg misoprostol showed a greater incidence of uterine tachysystole. This could be explained by the slow decline in plasma concentration of misoprostol after reaching maximum levels, resulting in abnormal uterine contractions (68). The American College of Obstetricians and Gynecologists recommends a lower dose of 25 μg misoprostol due to these potential uterine contractile abnormalities (69–71).

Our study had several limitations. Firstly, our meta-analysis included only eight studies with a limited sample size. Despite an extensive search strategy, few studies met the inclusion criteria for the meta-analysis. Additionally, this meta-analysis considered only publications in English, which could introduce bias and exclude pertinent studies published in other languages. Secondly, the dosages of misoprostol and dinoprostone varied across the studies, potentially affecting the interpretation of the results. Finally, the meta-analysis focused solely on the short-term effects of labor induction. Long-term outcomes, such as neonatal morbidity and maternal complications beyond the first few weeks postpartum, were not assessed.

These limitations should be considered when interpreting the findings of this meta-analysis and applying them to clinical practice. Future trials with larger sample sizes, standardized dosing protocols, and comprehensive outcome reporting are necessary to gain a clearer understanding of the efficacy and safety of misoprostol compared to dinoprostone for labor induction at term.

## Conclusion

In summary, our findings suggest that misoprostol and dinoprostone are comparably effective and safe for labor induction and misoprostol requires less oxytocin augmentation. The majority of analyzed outcomes exhibited low heterogeneity, indicating overall consistency among the included studies.

## Data Availability

The original contributions presented in the study are included in the article/Supplementary material, further inquiries can be directed to the corresponding author/s.
