# Supplementary material for: Efficacy and safety of misoprostol compared with dinoprostone for labor induction at term: an updated systematic review and meta-analysis of randomized controlled trials
Source: Front Med (Lausanne). 2024 Dec 9;11:1459793. doi: 10.3389/fmed.2024.1459793 (PMC11664862; doi:10.3389/fmed.2024.1459793)

**Supplementary material**

Supplementary Table 1: Search strategy of the study

Supplementary Table 2: Risk of bias assessment table with reasons

Supplementary Figure 1: Leave-one-out-analysis for vaginal delivery

Supplementary Table 1 – Search strategy of the study

|  |  |  |
| --- | --- | --- |
| **Search Strategy** | **Database** | **No. of Citation** |
| ("misoprostol"[MeSH Terms] OR "misoprostol"[All Fields] OR "misoprostol s"[All Fields] OR ("dinoprostone"[MeSH Terms] OR "dinoprostone"[All Fields] OR "dinoproston"[All Fields]) OR ("misoprostol"[MeSH Terms] OR "misoprostol"[All Fields] OR "cytotec"[All Fields] OR "misoprostol s"[All Fields]) OR ("arthrotec"[Supplementary Concept] OR "arthrotec"[All Fields] OR "arthrotec"[All Fields] OR "diclofenac"[MeSH Terms] OR "diclofenac"[All Fields] OR "misoprostol"[MeSH Terms] OR "misoprostol"[All Fields] OR "misoprostol s"[All Fields]) OR "Mifegymiso"[All Fields] OR ("dinoprostone"[MeSH Terms] OR "dinoprostone"[All Fields] OR "dinoproston"[All Fields] OR "prepidil"[All Fields]) OR ("dinoprostone"[MeSH Terms] OR "dinoprostone"[All Fields] OR "dinoproston"[All Fields] OR "prostin e2"[All Fields]) OR (("alprostadil"[MeSH Terms] OR "alprostadil"[All Fields] OR ("prostaglandin"[All Fields] AND "e1"[All Fields]) OR "prostaglandin e1"[All Fields]) AND ("analog"[All Fields] OR "analoge"[All Fields] OR "analoges"[All Fields] OR "analogic"[All Fields] OR "analogical"[All Fields] OR "analogizing"[All Fields] OR "analogous"[All Fields] OR "analogously"[All Fields] OR "analogs"[All Fields] OR "analogue"[All Fields] OR "analogues"[All Fields])) OR (("misoprostol"[MeSH Terms] OR "misoprostol"[All Fields] OR "misoprostol s"[All Fields]) AND ("intravaginal"[All Fields] OR "intravaginally"[All Fields])) OR (("dinoprostone"[MeSH Terms] OR "dinoprostone"[All Fields] OR "dinoproston"[All Fields]) AND ("intravaginal"[All Fields] OR "intravaginally"[All Fields]))) AND ("labor, induced"[MeSH Terms] OR ("labor"[All Fields] AND "induced"[All Fields]) OR "induced labor"[All Fields] OR ("labour"[All Fields] AND "induction"[All Fields]) OR "labour induction"[All Fields] OR (("child"[MeSH Terms] OR "child"[All Fields] OR "children"[All Fields] OR "child s"[All Fields] OR "children s"[All Fields] OR "childrens"[All Fields] OR "childs"[All Fields]) AND ("birth s"[All Fields] OR "birthed"[All Fields] OR "birthing"[All Fields] OR "parturition"[MeSH Terms] OR "parturition"[All Fields] OR "birth"[All Fields] OR "births"[All Fields])) OR (("induce"[All Fields] OR "induced"[All Fields] OR "inducer"[All Fields] OR "inducers"[All Fields] OR "induces"[All Fields] OR "inducibilities"[All Fields] OR "inducibility"[All Fields] OR "inducible"[All Fields] OR "inducing"[All Fields]) AND ("labor s"[All Fields] OR "labored"[All Fields] OR "laborer"[All Fields] OR "laborer s"[All Fields] OR "laborers"[All Fields] OR "laboring"[All Fields] OR "labors"[All Fields] OR "labour"[All Fields] OR "work"[MeSH Terms] OR "work"[All Fields] OR "labor"[All Fields] OR "labor, obstetric"[MeSH Terms] OR ("labor"[All Fields] AND "obstetric"[All Fields]) OR "obstetric labor"[All Fields] OR "laboured"[All Fields] OR "labourer"[All Fields] OR "labourers"[All Fields] OR "labouring"[All Fields] OR "labours"[All Fields])) OR ("birth s"[All Fields] OR "birthed"[All Fields] OR "birthing"[All Fields] OR "parturition"[MeSH Terms] OR "parturition"[All Fields] OR "birth"[All Fields] OR "births"[All Fields]) OR (("child"[MeSH Terms] OR "child"[All Fields] OR "children"[All Fields] OR "child s"[All Fields] OR "children s"[All Fields] OR "childrens"[All Fields] OR "childs"[All Fields]) AND ("bearing"[All Fields] OR "bearings"[All Fields])) OR ("Ceserian"[All Fields] AND ("deliveries"[All Fields] OR "delivery, obstetric"[MeSH Terms] OR ("delivery"[All Fields] AND "obstetric"[All Fields]) OR "obstetric delivery"[All Fields] OR "delivery"[All Fields])) OR ("cervical ripening"[MeSH Terms] OR ("cervical"[All Fields] AND "ripening"[All Fields]) OR "cervical ripening"[All Fields]) OR ("cesarean section"[MeSH Terms] OR ("cesarean"[All Fields] AND "section"[All Fields]) OR "cesarean section"[All Fields] OR "c section"[All Fields]) OR (("vagina"[MeSH Terms] OR "vagina"[All Fields] OR "vaginal"[All Fields] OR "vaginally"[All Fields] OR "vaginals"[All Fields] OR "vaginitis"[MeSH Terms] OR "vaginitis"[All Fields] OR "vaginitides"[All Fields]) AND ("deliveries"[All Fields] OR "delivery, obstetric"[MeSH Terms] OR ("delivery"[All Fields] AND "obstetric"[All Fields]) OR "obstetric delivery"[All Fields] OR "delivery"[All Fields]))) AND (("misoprostol"[MeSH Terms] OR "misoprostol"[All Fields] OR "misoprostol s"[All Fields] OR ("dinoprostone"[MeSH Terms] OR "dinoprostone"[All Fields] OR "dinoproston"[All Fields]) OR ("misoprostol"[MeSH Terms] OR "misoprostol"[All Fields] OR "cytotec"[All Fields] OR "misoprostol s"[All Fields]) OR ("arthrotec"[Supplementary Concept] OR "arthrotec"[All Fields] OR "arthrotec"[All Fields] OR "diclofenac"[MeSH Terms] OR "diclofenac"[All Fields] OR "misoprostol"[MeSH Terms] OR "misoprostol"[All Fields] OR "misoprostol s"[All Fields]) OR "Mifegymiso"[All Fields] OR ("dinoprostone"[MeSH Terms] OR "dinoprostone"[All Fields] OR "dinoproston"[All Fields] OR "prepidil"[All Fields]) OR ("dinoprostone"[MeSH Terms] OR "dinoprostone"[All Fields] OR "dinoproston"[All Fields] OR "prostin e2"[All Fields]) OR (("alprostadil"[MeSH Terms] OR "alprostadil"[All Fields] OR ("prostaglandin"[All Fields] AND "e1"[All Fields]) OR "prostaglandin e1"[All Fields]) AND ("analog"[All Fields] OR "analoge"[All Fields] OR "analoges"[All Fields] OR "analogic"[All Fields] OR "analogical"[All Fields] OR "analogizing"[All Fields] OR "analogous"[All Fields] OR "analogously"[All Fields] OR "analogs"[All Fields] OR "analogue"[All Fields] OR "analogues"[All Fields])) OR (("misoprostol"[MeSH Terms] OR "misoprostol"[All Fields] OR "misoprostol s"[All Fields]) AND ("intravaginal"[All Fields] OR "intravaginally"[All Fields])) OR (("dinoprostone"[MeSH Terms] OR "dinoprostone"[All Fields] OR "dinoproston"[All Fields]) AND ("intravaginal"[All Fields] OR "intravaginally"[All Fields]))) AND ("labor, induced"[MeSH Terms] OR ("labor"[All Fields] AND "induced"[All Fields]) OR "induced labor"[All Fields] OR ("labour"[All Fields] AND "induction"[All Fields]) OR "labour induction"[All Fields] OR (("child"[MeSH Terms] OR "child"[All Fields] OR "children"[All Fields] OR "child s"[All Fields] OR "children s"[All Fields] OR "childrens"[All Fields] OR "childs"[All Fields]) AND ("birth s"[All Fields] OR "birthed"[All Fields] OR "birthing"[All Fields] OR "parturition"[MeSH Terms] OR "parturition"[All Fields] OR "birth"[All Fields] OR "births"[All Fields])) OR (("induce"[All Fields] OR "induced"[All Fields] OR "inducer"[All Fields] OR "inducers"[All Fields] OR "induces"[All Fields] OR "inducibilities"[All Fields] OR "inducibility"[All Fields] OR "inducible"[All Fields] OR "inducing"[All Fields]) AND ("labor s"[All Fields] OR "labored"[All Fields] OR "laborer"[All Fields] OR "laborer s"[All Fields] OR "laborers"[All Fields] OR "laboring"[All Fields] OR "labors"[All Fields] OR "labour"[All Fields] OR "work"[MeSH Terms] OR "work"[All Fields] OR "labor"[All Fields] OR "labor, obstetric"[MeSH Terms] OR ("labor"[All Fields] AND "obstetric"[All Fields]) OR "obstetric labor"[All Fields] OR "laboured"[All Fields] OR "labourer"[All Fields] OR "labourers"[All Fields] OR "labouring"[All Fields] OR "labours"[All Fields])) OR ("birth s"[All Fields] OR "birthed"[All Fields] OR "birthing"[All Fields] OR "parturition"[MeSH Terms] OR "parturition"[All Fields] OR "birth"[All Fields] OR "births"[All Fields]) OR (("child"[MeSH Terms] OR "child"[All Fields] OR "children"[All Fields] OR "child s"[All Fields] OR "children s"[All Fields] OR "childrens"[All Fields] OR "childs"[All Fields]) AND ("bearing"[All Fields] OR "bearings"[All Fields])) OR ("Ceserian"[All Fields] AND ("deliveries"[All Fields] OR "delivery, obstetric"[MeSH Terms] OR ("delivery"[All Fields] AND "obstetric"[All Fields]) OR "obstetric delivery"[All Fields] OR "delivery"[All Fields])) OR ("cervical ripening"[MeSH Terms] OR ("cervical"[All Fields] AND "ripening"[All Fields]) OR "cervical ripening"[All Fields]) OR ("cesarean section"[MeSH Terms] OR ("cesarean"[All Fields] AND "section"[All Fields]) OR "cesarean section"[All Fields] OR "c section"[All Fields]) OR (("vagina"[MeSH Terms] OR "vagina"[All Fields] OR "vaginal"[All Fields] OR "vaginally"[All Fields] OR "vaginals"[All Fields] OR "vaginitis"[MeSH Terms] OR "vaginitis"[All Fields] OR "vaginitides"[All Fields]) AND ("deliveries"[All Fields] OR "delivery, obstetric"[MeSH Terms] OR ("delivery"[All Fields] AND "obstetric"[All Fields]) OR "obstetric delivery"[All Fields] OR "delivery"[All Fields])))) | PUBMED | 3165 |
| (Misoprostol OR Dinoprostone OR Cytotec OR Arthrotec OR Mifegymiso OR Prepidil OR Prostin E2 OR Prostaglandin E1 Analogue OR Misoprostol intravaginally OR Dinoprostone intravaginally) AND (labour induction OR Child birth OR inducing labor OR birthing OR child bearing OR Ceserian Delivery OR Cervical ripening OR C-section OR Vaginal Delivery) | GOOGLE SCHOLAR | 1700 |
| (Misoprostol OR Dinoprostone OR Cytotec OR Arthrotec OR Mifegymiso OR Prepidil OR Prostin E2 OR Prostaglandin E1 Analogue OR Misoprostol intravaginally OR Dinoprostone intravaginally) AND (labour induction OR Child birth OR inducing labor OR birthing OR child bearing OR Ceserian Delivery OR Cervical ripening OR C-section OR Vaginal Delivery)  (Misoprostol OR Dinoprostone OR Cytotec OR Arthrotec OR Mifegymiso OR Prepidil OR Prostin E2 OR Prostaglandin E1 Analogue OR Misoprostol intravaginally OR Dinoprostone intravaginally) AND (labour induction OR Child birth OR inducing labor OR birthing OR child bearing OR Ceserian Delivery OR Cervical ripening OR C-section OR Vaginal Delivery) | COCHRANE LIBRARY  EBSCO | 1983  772 |

Supplementary Figure 1: Leave-one-out-analysis for vaginal delivery


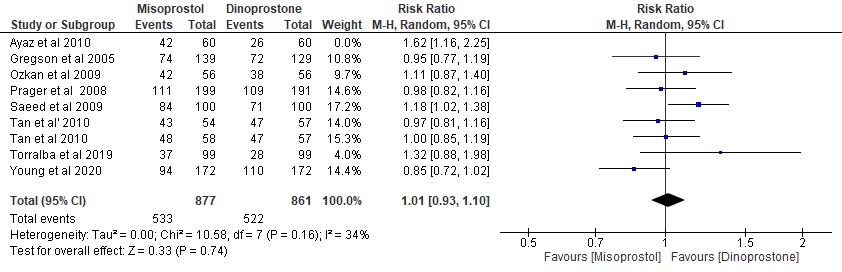

Supplement: Supplementary file 1 [file Table_1.DOCX]
